# Supplementary material for: Individual differences in information-seeking
Source: Nat Commun. 2021 Dec 3;12:7062. doi: 10.1038/s41467-021-27046-5 (PMC8642448; doi:10.1038/s41467-021-27046-5)
Supplement: Supplementary file 1 — Supplementary Information [file 41467_2021_27046_MOESM1_ESM.pdf]

Supplementary Information

Supplementary Methods

Supplementary Table 1. *Experiment 1: Average scores of ratings and their correlations.*

Mean of task variables (standard deviation).
(***p<0.001, **p <0.01, *p<0.05 (two-sided): one-sample t-test **comparing mean to mid-point of their scale**).

	Information-Seeking Choice *(-3 to +3)*	Usefulness *(-3 to +3)*	Thought Frequency *(-3 to +3)*	Feelings to Know *(-3 to +3)*	Feelings Never to Know *(-3 to +3)*	Feelings to Know - Feelings Never to Know *(-6 to +6)*	Expected rating of others ***(Negative traits reverse scored; -3 to +3)***	Confidence in estimation *(-3 to +3)*
Task Ratings Mean (SD)	0.43** (1.30)	0.60*** (1.19)	0.15 (0.85)	0.73*** (0.89)	0.06 (0.92)	0.67*** (1.35)	0.95*** (1.07)	1.55*** (0.78)

Mean Pearson R between variables calculated for each participant and then averaged across participants (standard deviation)**.**
(***p<0.001, **p <0.01, *p<0.05 (two-sided): one-sample t-test comparing the mean R across subjects to zero).

	Information-Seeking Choice	Usefulness	Thought Frequency	Feelings to Know	Feelings Never to Know	Feelings to Know - Feelings Never to Know	Expected rating	Confidence in estimation
Information-Seeking Choice Mean (SD)	-							
Usefulness Mean (SD)	0.21*** (0.23)	-						
Thought Frequency Mean (SD)	0.21*** (0.24)	0.21*** (0.23)	-					
Feelings to Know Mean (SD)	0.23*** (0.23)	0.31*** (0.29)	0.16*** (0.27)	-				
Feelings Never to Know Mean (SD)	-0.13*** (0.23)	-0.24*** (0.31)	-0.10** (0.25)	-0.25*** (0.41)	-			
Feelings to Know - Feelings Never to Know Mean (SD)	0.23*** (0.24)	0.34*** (0.29)	0.17*** (0.26)	0.82*** (0.20)	-0.70*** (0.21)	-		
Expected rating of others Mean (SD)	0.19*** (0.26)	0.15*** (0.31)	0.07** (0.22)	0.40*** (0.32)	-0.21*** (0.28)	0.38*** (0.31)	-	
Confidence in estimation Mean (SD)	0.05 (0.22)	0.02 (0.38)	0.10*** (0.20)	0.25*** (0.29)	-0.02 (0.29)	0.18*** (0.29)	0.29*** (0.31)	-

Information-Seeking Choice Rating: -3 ('*definitely don't want to know'*) to +3 ('*definitely want to know'*); **Usefulness:** -3 ('*not useful*') to +3 (*very useful*); **Thought Frequency:** -3 ('*never*') to +3 ('very *often'*); **Feelings to Know:** -3 ('*very bad')* to +3 ('*very good'*); **Feelings Not to Know:** -3 ('*very*

bad') to (+3 '*very good*'); **Expected Rating of Others:** -3 (*'not at all this trait')* to +3 (*'very much this trait';* scores were reversed for negative valanced stimuli); **Confidence in Estimation:** -3 ('*not certain')* to +3 (*'very certain'*).

Supplementary Table 2. *Experiment 2, Time 1: Average scores of ratings and their correlations.*

Mean of task variables (standard deviation).
(***p<0.001, **p <0.01, *p<0.05 (two-sided): one-sample t-test **comparing mean to mid-point of their scale**).

	Information-Seeking Choice ***(-3 to +3)***	Usefulness ***(-3 to +3)***	Thought Frequency ***(-3 to +3)***	Feelings to Know ***(-3 to +3)***	Feelings Never to Know ***(-3 to +3)***	Feelings to Know - Feelings Never to Know ***(-6 to +6)***	Expected rating of others ***(Negative traits reverse scored; -3 to +3)***	Confidence in estimation ***(-3 to +3)***
Task Ratings Mean (SD)	1.17*** (1.02)	0.75*** (1.01)	0.53*** (0.81)	0.67*** (0.72)	0.00 (0.86)	0.68*** (1.16)	0.93*** (0.66)	1.20*** (0.82)

Mean Pearson R between variables calculated for each participant and then averaged across participants (standard deviation**).**
(***p<0.001, **p <0.01, *p<0.05 (two-sided): one-sample t-test comparing the mean R across subjects to zero).

	Information-Seeking Choice	Usefulness	Thought Frequency	Feelings to Know	Feelings Never to Know	Feelings to Know - - Feelings Never to Know	Expected rating of others	Confidence in estimation
Information-Seeking Choice Mean (SD)	-							
Usefulness Mean (SD)	0.165*** (0.23)	-						
Thought Frequency Mean (SD)	0.12*** (0.21)	0.20*** (0.22)	-					
Feelings to Know Mean (SD)	0.18*** (0.22)	0.30*** (0.32)	0.09*** (0.24)	-				
Feelings Never to Know Mean (SD)	-0.12*** (0.22)	-0.23*** (0.31)	-0.08*** (0.24)	-0.20*** (0.40)	-			
Feelings to Know - Feelings Never to Know Mean (SD)	0.19*** (0.22)	0.32*** (0.30)	0.10*** (0.23)	0.83*** (0.16)	-0.68*** (0.24)	-		
Expected rating of others Mean (SD)	0.20*** (0.23)	0.15*** (0.26)	-0.02 (0.21)	0.35*** (0.29)	-0.14*** (0.27)	0.32*** (0.29)	-	
Confidence in estimation Mean (SD)	0.07*** (0.20)	0.02 (0.33)	0.11*** (0.21)	0.19*** (0.27)	0.01 (0.25)	0.13*** (0.27)	0.23*** (0.27)	-

Information-Seeking Choice Rating: -3 ('*definitely don't want to know'*) to +3 ('*definitely want to know'*); **Usefulness:** -3 ('*not useful*') to +3 (*very useful*); **Thought Frequency:** -3 ('*never*') to +3 ('very *often'*); **Feelings to Know:** -3 ('*very bad')* to +3 ('*very good'*); **Feelings Not to Know:** -3 ('*very bad')* to (+3 '*very good'*); **Expected Rating of Others:** -3 (*'not at all this trait')* to +3 (*'very much this trait';* scores were reversed for negative valanced stimuli); **Confidence in Estimation:** -3 ('*not certain')* to +3 (*'very certain'*).

Supplementary Table 3. *Experiment 2, Time 2: Average scores of ratings and their correlations.*

Mean of task variables (standard deviation).
(***p<0.001, **p <0.01, *p<0.05 (two-sided): one-sample t-test **comparing mean to mid-point of their scale**).

	Information-Seeking Choice *(-3 to +3)*	Usefulness *(-3 to +3)*	Thought Frequency *(-3 to +3)*	Feelings to Know *(-3 to +3)*	Feelings Never to Know *(-3 to +3)*	Feelings to Know - Feelings Never to Know *(-6 to +6)*	Expected rating of others ***(Negative traits reverse scored; -3 to +3)***	Confidence in estimation *(-3 to +3)*
Task Ratings Mean (SD)	0.74*** (0.82)	0.56*** (0.93)	0.41*** (0.78)	0.52*** (0.65)	0.08 (0.75)	0.43*** (1.05)	0.72*** (0.67)	1.03*** (0.77)

Mean Pearson R between variables calculated for each participant and then averaged across participants (standard deviation)**.**
(***p<0.001, **p <0.01, *p<0.05 (two-sided): one-sample t-test comparing the mean R across subjects to zero).

	Information-Seeking Choice	Usefulness	Thought Frequency	Feelings to Know	Feelings Never to Know	Feelings to Know - Feelings Never to Know	Expected rating of others	Confidence in estimation
Information-Seeking Choice Mean (SD)	-							
Usefulness Mean (SD)	0.18*** (0.20)	-						
Thought Frequency Mean (SD)	0.16*** (0.22)	0.21*** (0.23)	-					
Feelings to Know Mean (SD)	0.19*** (0.23)	0.30*** (0.31)	0.12*** (0.25)	-				
Feelings Never to Know Mean (SD)	-0.13*** (0.21)	-0.18*** (0.32)	-0.08*** (0.23)	-0.17*** (0.42)	-			
Feelings to Know - Feelings Never to Know Mean (SD)	0.20*** (0.21)	0.31*** (0.30)	0.12*** (0.24)	0.82** (0.17)	-0.67*** (0.23)	-		
Expected rating of others Mean (SD)	0.21*** (0.25)	0.14*** (0.28)	0.00 (0.23)	0.35*** (0.29)	-0.11*** (0.27)	0.31*** (0.29)	-	
Confidence in estimation Mean (SD)	0.02 (0.19)	0.01 (0.34)	0.11*** (0.21)	0.17*** (0.26)	0.04 (0.23)	0.11*** (0.24)	0.20** (0.27)	-

Information-Seeking Choice Rating: -3 (‘*definitely don't want to know*’) to +3 (‘*definitely want to know*’); **Usefulness:** -3 (‘*not useful*‘) to +3 (*very useful*); **Thought Frequency:** -3 (‘*never*‘) to +3 (‘very *often*’); **Feelings to Know:** -3 (‘*very bad’)* to +3 (‘*very good*’); **Feelings Not to Know:** -3 (‘*very bad’)* to (+3 ‘*very good’*); **Expected Rating of Others:** -3 (*‘not at all this trait’)* to +3 (*‘very much this trait’;* scores were reversed for negative valanced stimuli); **Confidence in Estimation:** -3 (‘*not certain ’)* to +3 (*‘very certain ’*).

Supplementary Table 4. *Experiment 3, Time 1: Average scores of ratings and their correlations.*

Mean of task variables (standard deviation).
(***p<0.001, **p <0.01, *p<0.05 (two-sided): one-sample t-test **comparing mean to mid-point of their scale**).

	Information-Seeking Choice *(-3 to +3)*	Usefulness *(-3 to +3)*	Thought Frequency *(-3 to +3)*	Feelings to Know *(-3 to +3)*	Feelings Never to Know *(-3 to +3)*	Feelings to Know - Feelings Never to Know *(-6 to +6)*	Confidence in estimation *(-3 to +3)*
Task Ratings Mean (SD)	1.12*** (0.90)	1.14*** (0.95)	0.67*** (1.04)	0.99*** (0.93)	-0.34*** (0.96)	1.33*** (1.39)	0.42*** (1.07)

Mean Pearson R between variables calculated for each participant and then averaged across participants (standard deviation)**.**
(***p<0.001, **p <0.01, *p<0.05 (two-sided): one-sample t-test comparing the mean R across subjects to zero).

	Information-Seeking Choice	Usefulness	Thought Frequency	Feelings to Know	Feelings Never to Know	Feelings to Know - Feelings Never to Know	Confidence in estimation
Information-Seeking Choice Mean (SD)	-						
Usefulness Mean (SD)	0.37*** (0.29)	-					
Thought Frequency Mean (SD)	0.27*** (0.24)	0.30*** (0.25)	-				
Feelings to Know Mean (SD)	0.25*** (0.25)	0.37*** (0.36)	0.18*** (0.25)	-			
Feelings Never to Know Mean (SD)	-0.23*** (0.22)	-0.33*** (0.28)	-0.22*** (0.27)	-0.26*** (0.34)	-		
Feelings to Know - Feelings Never to Know Mean (SD)	0.29*** (0.24)	0.42*** (0.31)	0.23*** (0.24)	0.80*** (0.16)	-0.75*** (0.17)	-	
Confidence in estimation Mean (SD)	0.11*** (0.16)	0.17*** (0.26)	0.19*** (0.22)	0.10*** (0.24)	-0.05** (0.21)	0.09*** (0.20)	-

Information-Seeking Choice Rating: -3 (‘*definitely don’t want to know*’) to +3 (‘*definitely want to know*’); **Usefulness:** -3 (‘*not useful*’) to +3 (*very useful*); **Thought Frequency:** -3 (‘*never*’) to +3 (‘very *often*’); **Feelings to Know:** -3 (‘*very bad’)* to +3 (‘*very good*’); **Feelings Not to Know:** -3 (‘*very bad’)* to (+3 ‘*very good*’); **Confidence in Estimation:** -3 (‘*not certain’)* to +3 (‘*very certain*’).

Supplementary Table 5. *Experiment 3, Time 2: Average scores of ratings and their correlations.*

Mean of task variables (standard deviation).
(***p<0.001, **p <0.01, *p<0.05 (two-sided): one-sample t-test **comparing mean to mid-point of their scale**).

	Information-Seeking Choice *(-3 to +3)*	Usefulness *(-3 to +3)*	Thought Frequency *(-3 to +3)*	Feelings to Know *(-3 to +3)*	Feelings Never to Know *(-3 to +3)*	Feelings to Know - Feelings Never to Know *(-6 to +6)*	Confidence in estimation *(-3 to +3)*
Task Ratings Mean (SD)	0.89*** (1.00)	0.79*** (1.12)	0.28* (1.12)	0.71*** (1.00)	-0.33** (0.95)	1.04*** (1.40)	0.09 (1.11)

Mean Pearson R between variables calculated for each participant and then averaged across participants (standard deviation**).**
(***p<0.001, **p <0.01, *p<0.05 (two-sided): one-sample t-test comparing the mean R across subjects to zero).

	Information-Seeking Choice	Usefulness	Thought Frequency	Feelings to Know	Feelings Never to Know	Feelings to Know - Feelings Never to Know	Confidence in estimation
Information-Seeking Choice Mean (SD)	-						
Usefulness Mean (SD)	0.44*** (0.29)	-					
Thought Frequency Mean (SD)	0.37*** (0.23)	0.43*** (0.25)	-				
Feelings to Know Mean (SD)	0.28*** (0.24)	0.42*** (0.34)	0.26*** (0.29)	-			
Feelings Never to Know Mean (SD)	-0.30*** (0.25)	-0.32*** (0.40)	-0.28*** (0.30)	-0.29*** (0.42)	-		
Feelings to Know - Feelings Never to Know Mean (SD)	0.34*** (0.24)	0.42*** (0.33)	0.30*** (0.27)	0.82*** (0.17)	-0.75*** (0.19)	-	
Confidence in estimation Mean (SD)	0.14*** (0.17)	0.18*** (0.29)	0.25*** (0.19)	0.10** (0.30)	-0.07* (0.26)	0.09*** (0.25)	-

Information-Seeking Choice Rating: -3 ('*definitely don't want to know*') to +3 ('*definitely want to know*'); **Usefulness:** -3 ('*not useful*') to +3 (*very useful*); **Thought Frequency:** -3 ('*never*') to +3 ('very *often*'); **Feelings to Know:** -3 ('*very bad')* to +3 ('*very good*'); **Feelings Not to Know:** -3 ('*very bad')* to (+3 '*very good*'); **Confidence in Estimation:** -3 ('*not certain')* to +3 ('*very certain*').

Supplementary Table 6. *Experiment 4: Average scores of ratings and their correlations.*

Mean of task variables (standard deviation).
(***p<0.001, **p <0.01, *p<0.05 (two-sided): one-sample t-test **comparing mean to mid-point of their scale**).

	Information-Seeking Choice<br>***(-3 to +3)***	Usefulness<br>***(-3 to +3)***	Thought Frequency<br>***(-3 to +3)***	Feelings to Know<br>***(-3 to +3)***	Feelings Never to Know<br>***(-3 to +3)***	Feelings to Know - Feelings Never to Know<br>***(-6 to +6)***	Expectations ***(Negative stimuli reverse scored; -3 to +3)***	Confidence in estimation<br>***(-3 to +3)***
Task Ratings<br>Mean (SD)	1.47***<br>(0.99)	1.13***<br>(0.88)	-0.15<br>(0.90)	0.89***<br>(0.84)	-0.29***<br>(0.77)	1.13***<br>(1.21)	0.40***<br>(0.69)	0.80***<br>(0.99)

Mean Pearson R between variables calculated for each participant and then averaged across participants (standard deviation)**.**
(***p<0.001, **p <0.01, *p<0.05 (two-sided): one-sample t-test comparing the mean R across subjects to zero).

	Information-Seeking Choice	Usefulness	Thought Frequency	Feelings to Know	Feelings Never to Know	Feelings to Know - Feelings Never to Know	Expected rating	Confidence in estimation
Information-Seeking Choice<br>Mean (SD)	-							
Usefulness<br>Mean (SD)	0.40***<br>(0.28)	-						
Thought Frequency<br>(SD)	0.16***<br>(0.21)	0.21***<br>(0.24)	-					
Feelings to Know<br>Mean (SD)	0.26***<br>(0.27)	0.38***<br>(0.36)	0.22***<br>(0.25)	-				
Feelings Never to Know<br>Mean (SD)	-0.22***<br>(0.24)	-0.30***<br>(0.33)	-0.19***<br>(0.26)	-0.25***<br>(0.36)	-			
Feelings to Know - Feelings Never to Know<br>Mean (SD)	0.29***<br>(0.26)	0.41***<br>(0.33)	0.26***<br>(0.26)	0.84***<br>(0.13)	-0.71***<br>(0.23)	-		
Expectations<br>Mean (SD)	0.00<br>(0.18)	0.02<br>(0.22)	-0.27***<br>(0.23)	0.02<br>(0.22)	0.09***<br>(0.21)	-0.04<br>(0.21)	-	
Confidence in estimation<br>Mean (SD)	-0.02<br>(0.21)	-0.01<br>(0.25)	0.11****<br>(0.24)	0.06*<br>(0.26)	0.04<br>(0.21)	0.02<br>(0.25)	0.04<br>(0.28)	-

Information-Seeking Choice Rating: -3 ('*definitely don't want to know'*) to +3 ('*definitely want to know'*); **Usefulness:** -3 ('*not useful*') to +3 (*very useful*); **Thought Frequency:** -3 ('*never*') to +3 ('very *often'*); **Feelings to Know:** -3 ('*very bad')* to +3 ('*very good'*); **Feelings Not to Know:** -3 ('*very bad')* to (+3 '*very good'*); **Expectations:** -3 (*'not at all this trait')* to +3 (*'very much this trait';* scores were reversed for negative valanced stimuli); **Confidence in Estimation:** -3 ('*not certain')* to +3 (*'very certain'*).

Supplementary Experiment (Experiment 5)

Testing additional ratings. We ran a fifth study (N= 48) to examine if additional ratings explain information-seeking when competing for variance with the ratings described in the main text. Forty-five participants who passed the attention checks and for whom data variability allowed the generation of all beta coefficients were included in the analysis.

The procedure was exactly as in Experiment 1 except that three additional ratings were included:

(1) **Distinctiveness.** One may hypothesize that individuals would be more interested in receiving information about traits they believe make them unique relative to others. We thus asked subjects to rate 'how much do you differ from others on *this trait*?' on a scale from -3 (Not Different) to +3 (Very Different)

(2) **Sense Making.** One may hypothesize that individuals would be more interested in receiving information that will help them make sense of things that happened in their lives. We thus asked subjects 'if you knew how others rated you on *this trait*, would it help you make sense of things that happened in your life?' on a scale from -3 (Not at All) to +3 (Very Much)

(3) **Recency.** One may hypothesize that individuals would be more interested in receiving information about topics they had contemplated lately. We thus asked subjects 'before today, when was the last time you thought of whether others view you on *this trait*?' on a scale from -3 (Never) to +3 (Last 24-Hours).

We then entered the three ratings above into a linear mixed effect model predicting information-seeking choice along with the three questions from our hypothesized model as well as participants' confidence in their expectations of what the information would reveal. A model with random effects to item and subject did not converge. In line with recommendations[63,64] we reduced the random effect structure until the model was able to converge. This occurred when random effects were assigned to subject and not item. The results revealed that participants were more likely to seek information for topics they thought of often (Cognitive Utility $\beta = 0.102 \pm 0.032$ (SE), $t(65.84) = 3.228$, $p = 0.002$), when they expected to feel better after knowing than not knowing (Hedonic Utility $\beta = 0.116 \pm 0.044$ (SE), $t(31.78) = 2.625$, $p = 0.013$), as well as tended to want information more when they expected information to be useful (Instrumental Utility $\beta = 0.071 \pm 0.039$ (SE), $t(23.41) = 1.810$, $p = 0.08$) and for topics they thought off recently (Recency: $\beta = 0.084 \pm 0.047$ (SE), $t(34.71) = 1.786$, $p = 0.08$). Confidence ($\beta = 0.045 \pm 0.046$ (SE), $t(37.34) = 0.974$, $p = 0.336$), Distinctiveness ($\beta = 0.033 \pm 0.034$ (SE), $t(48.80) = 0.950$, $p = 0.347$) and Sense Making ($\beta = -0.018 \pm 0.045$ (SE), $t(36.69) = -0.389$, $p = 0.7$) were not significant predictors (**Supplementary Figure 1**).

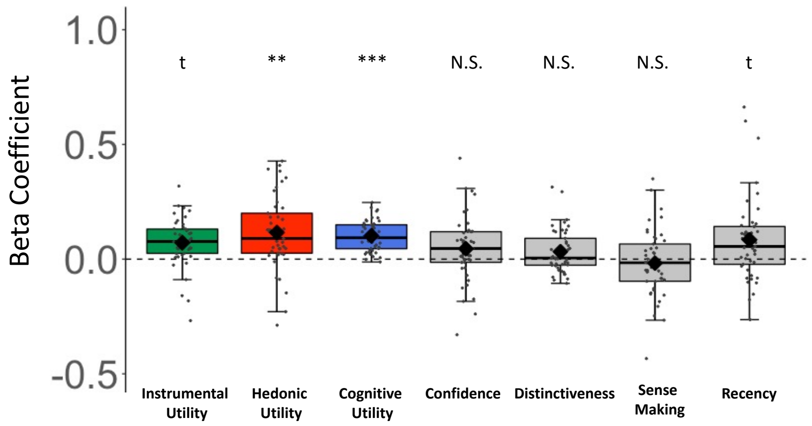

Supplementary Figure 1: Information-Seeking Motives. Plotted are beta coefficients from a linear mixed effects model predicting information-seeking (N = 45 subjects), which shows participants want information more when they expect information to make them feel better than ignorance (Hedonic Utility), and for topics they think of often (Cognitive Utility), as well as tend to want information more when its Instrumental Utility is high and for topics they thought of recently (Recency). The horizontal lines indicate median values, boxes indicate 25–75% interquartile range and whiskers indicate 1.5 × interquartile range; individual scores are shown as dots. Distinctiveness (i.e., how much one differs from others on a trait), Sense Making (i.e., whether knowing would help make sense of things that happened in one's life), and Confidence, were not significant predictors of information-seeking. *** = P <0.001.**P <0.01, t = trend, N.S. = not significant (two-sided).

Information-seeking Task

Experiments 1 & 2

(a) Block 1: Measure of information-seeking (40 trials)

Would you like to know how your family/friends rated you on being: **Kind**?

0	0	0	0	0	0
Definitely Don't Want to Know	Don't Want to Know	Somewhat Don't Want to Know	Somewhat Want to Know	Want to Know	Definitely Want to Know

Would you like to know how your family/friends rated you on being: **Mean**?

0	0	0	0	0	0
Definitely Don't Want to Know	Don't Want to Know	Somewhat Don't Want to Know	Somewhat Want to Know	Want to Know	Definitely Want to Know

(b) Block 2: Measure of information-seeking (40 trials)

With regard to being **Kind:**

What rating do you think your family and friends will give you?

0	0	0	0	0	0	0
Not at all this trait (-3)	(-2)	(-1)	(0)	(1)	(2)	Very much this trait (3)

How **CERTAIN** are you about your estimate?

0	0	0	0	0	0	0
Not Certain (-3)	(-2)	(-1)	(0)	(1)	(2)	Very Certain (3)

How **USEFUL** would it be to know how your friends/family have rated you?

0	0	0	0	0	0	0
Not Useful (-3)	(-2)	(-1)	(0)	(1)	(2)	Very Useful (3)

How would you **FEEL** if you got to find out how you were rated?

0	0	0	0	0	0	0
Very Bad (-3)	(-2)	(-1)	(0)	(1)	(2)	Very Good (3)

How would you **FEEL** if you **NEVER** get to find out how you were rated?

0	0	0	0	0	0	0
Very Bad (-3)	(-2)	(-1)	(0)	(1)	(2)	Very Good (3)

How **OFTEN** do you think about **Kindness**?

0	0	0	0	0	0	0
Never (-3)	(-2)	(-1)	(0)	(1)	(2)	Very Often (3)

Experiment 3

(c) Block 1: Measure of information-seeking (40 trials)

Do you want to know what the Gross Domestic Profit is?

0	0	0	0	0	0
Definitely Don't Want to Know	Don't Want to Know	Somewhat Don't Want to Know	Somewhat Want to Know	Want to Know	Definitely Want to Know

Do you want to know what your health expenses are?

0	0	0	0	0	0
Definitely Don't Want to Know	Don't Want to Know	Somewhat Don't Want to Know	Somewhat Want to Know	Want to Know	Definitely Want to Know

(d) Block 2: Measure of information-seeking (40 trials)

For the question about: **what the Gross Domestic Profit is?**

What do you think the answer is?

0	0	0
Low	Average	High

How **CERTAIN** are you in your answer?

0	0	0	0	0	0	0
Not Certain (-3)	(-2)	(-1)	(0)	(1)	(2)	Very Certain (3)

How **USEFUL** would it be to know the answer to this question?

0	0	0	0	0	0	0
Not Useful (-3)	(-2)	(-1)	(0)	(1)	(2)	Very Useful (3)

How would you **FEEL** if you got to find out the answer?

0	0	0	0	0	0	0
Very Bad (-3)	(-2)	(-1)	(0)	(1)	(2)	Very Good (3)

How would you **FEEL** if you **NEVER** get to find out the answer?

0	0	0	0	0	0	0
Very Bad (-3)	(-2)	(-1)	(0)	(1)	(2)	Very Good (3)

How **OFTEN** do you think about **Gross Domestic Profit**?

0	0	0	0	0	0	0
Never (-3)	(-2)	(-1)	(0)	(1)	(2)	Very Often (3)

Experiment 4

(e) Block 1: Measure of information-seeking (40 trials)

Would you like to know if you have a gene that increases your likelihood of: **Alzheimer's disease?**?

0	0	0	0	0	0
Definitely Don't Want to Know	Don't Want to Know	Somewhat Don't Want to Know	Somewhat Want to Know	Want to Know	Definitely Want to Know

Would you like to know if you have a gene that increases your likelihood of: **Good Memory?**

0	0	0	0	0	0
Definitely Don't Want to Know	Don't Want to Know	Somewhat Don't Want to Know	Somewhat Want to Know	Want to Know	Definitely Want to Know

(f) Block 2: Measure of information-seeking (40 trials)

With regard to the gene that increases the likelihood for: **Alzheimer's disease**

How **LIKELY** is it that you carry this gene?

0	0	0	0	0	0	0
Not Likely (-3)	(-2)	(-1)	(0)	(1)	(2)	Very Likely (3)

How **CERTAIN** are you about your estimate?

0	0	0	0	0	0	0
Not Certain (-3)	(-2)	(-1)	(0)	(1)	(2)	Very Certain (3)

How **USEFUL** would it be to know whether or not you carry this gene?

0	0	0	0	0	0	0
Not Useful (-3)	(-2)	(-1)	(0)	(1)	(2)	Very Useful (3)

How would you **FEEL** if you got to find out whether or not you carry this gene?

0	0	0	0	0	0	0
Very Bad (-3)	(-2)	(-1)	(0)	(1)	(2)	Very Good (3)

How would you **FEEL** if you **NEVER** get to find out whether or not you carry this gene?

0	0	0	0	0	0	0
Very Bad (-3)	(-2)	(-1)	(0)	(1)	(2)	Very Good (3)

How **OFTEN** do you think about: **Alzheimer's disease?**

0	0	0	0	0	0	0
Never (-3)	(-2)	(-1)	(0)	(1)	(2)	Very Often (3)

Supplementary Figure 2: Information-Seeking Task. (a) For Experiments 1 & 2, participants were asked to imagine that their family/friends have rated them on 40 different attributes. They then indicated whether they would like to know how they have been rated (e.g., on being kind) from '*definitely don't want to know'* to '*definitely want to know'.* **(c)** For experiment 3, participants were asked to indicate whether they wanted to know different 40 pieces of information related to finance (e.g., what the Gross Domestic Profit is) from '*definitely don't want to know'* to '*definitely want to know'.* **(e)** For experiment 4, participants were asked to imagine that we had information about their genetic makeup and asked whether they wanted to know 40 pieces of information related to Health (*"Would you like to know if you have a gene that increases your likelihood of Alzheimer's disease?"*) from '*definitely don't want to know'* to '*definitely want to know'.* **(b, d)** Next, participants provided the following ratings for each stimulus (self-paced): (i) Their expectations regarding how useful each piece of information would be from '*not useful* ' to '*very useful'*; (ii) How they expect to feel if the rating was revealed to them from '*very bad'* to '*very good'*, and how they expect to feel if the rating was never revealed to them from '*very bad'* to '*very good'*.; (iii) How often they think about the topic in question from '*never* 'to 'very *often'*. Each question is displayed separately for each stimulus. Participants were also asked in Experiment 1 & 2 to indicate for each attribute (i) what rating they think their family and friends will give them and (ii) how certain

they are in that. In Experiment 3 they were asked to indicate (i) what they thought the answer was (for each stimulus the scale was different for this question and for some stimuli this was an open-ended question) and (ii) how certain they are in their answer. In Experiment 4, participants indicated their expectations of how likely it is that they carry the gene (from *-3 'not likely'* to *+3 'very likely', e.g.,* how likely is it that you carry this gene?). Finally, participants indicated their confidence in what they expected the information would reveal (from -3 '*not certain'* to +3 *'very certain'*). In Experiment 1 & 2, participants filled in questionnaires assessing mental health[22–30] following the task.

Supplementary Table 7. Experiments 1 & 2: Correlations (partial R coefficients) between the weight subjects assign to motives of information-seeking and scores on specific psychopathology questionnaires.

Experiment 1 ***Psychopathology Questionnaires***	**Instrumental Utility (β_1)**	**Hedonic Utility (β_2)**	**Cognitive Utility (β_3)**
Depression	-0.111	0.083	-0.011
Anxiety	-0.093	0.137	-0.197
Apathy	-0.089	-0.037	-0.146
OCD	-0.057	0.068	-0.115
Social Anxiety	0.086	0.116	-0.276*
Alcohol Use Disorder	0.024	-0.082	-0.113
Impulsivity	-0.040	-0.047	0.010
Schizotypy	-0.229*	0.080	-0.108
Eating Disorder	-0.274*	0.087	-0.146

Experiment 2[1] ***Psychopathology Questionnaires***	**Instrumental Utility (β_1)**	**Hedonic Utility (β_2)**	**Cognitive Utility (β_3)**
Depression	0.088	0.249***	-0.138
Anxiety	0.086	0.209*	-0.132
Apathy	0.061	0.236**	-0.269***
OCD	0.078	-0.012	-0.177*
Social Anxiety	0.088	0.190*	-0.204
Alcohol Use Disorder	0.169	-0.083	-0.065
Impulsivity	0.058	-0.061	-0.355***
Schizotypy	0.106	0.197*	-0.263***
Eating Disorder	0.050	-0.059	-0.177

*Bonferroni corrected: ***p< 0.006, No correction: **p<0.01, *p<0.05* (two-sided).
Displayed are the partial R coefficients controlling for gender and age.
The following questionnaires were used to asses psychopathology: **Depression** = Self-Rating Depression Scale[23]; **Anxiety** = State-Trait Anxiety Inventory[24]; **Apathy** = Apathy Evaluation Scale[26]; **OCD** = Obsessive-Compulsive Inventory – Revised[22]; **Social Anxiety** = Liebowitz Social Anxiety Scale[30]; **Alcohol Use Disorder** = Alcohol Use Disorder Identification Test[25]; **Impulsivity** = Barratt Impulsivity Scale[28]; **Schizotypy** = Short Scales for Measuring Schizotypy[29] ;**Eating Disorder** = EAT-26[27].
[1]Data displayed for Experiment 2 are psychopathology scores correlated with the mean beta coefficient for each motive over Time 1 and Time 2.

Supplementary Table 8. *Experiments 1 - 4: AIC scores for all models.*

Model (AIC Score)	Experiment 1	Experiment 2, Time 1	Experiment 2, Time 2	Experiment 3, Time 1	Experiment 3, Time 2	Experiment 4
Instrumental + Hedonic + Cognitive	**11066.38**	**24863.27**	**17242.26**	**17595.42**	**11625.84**	**15456.62**
Instrumental + Hedonic + Cognitive + Confidence	11070.74	24869.11	17247.84	17608.72	11639.83	15463.2
Hedonic + Cognitive + Confidence	11107.96	24907.92	17260.81	17874.96	11922.91	15842.82
Hedonic + Cognitive	11109.39	24905.13	17257.45	17885.18	11911.51	15834.96
Instrumental + Cognitive + Confidence	11122.51	25020.58	17342.45	17663.99	11663.03	15568.16
Instrumental +Hedonic + Confidence	11186.01	24914.37	17303.71	17707.74	11756.56	15523.26
Instrumental + Cognitive	11301.75	25067.15	17405.32	17776	11801.1	15654.25
Instrumental + Hedonic	11183.4	24915.38	17301.11	17750.06	11750.34	15517.1
Cognitive + Confidence	11200.15	25148.86	17410.02	18090.35	12062.3	16135.68
Cognitive	11215.41	25157.28	17404.61	18078.92	12051.43	16134.31
Hedonic + Confidence	11247.08	24977.47	17329.6	18107.07	12179.93	15966.8
Hedonic	11251.14	24982.45	17329.49	18111.19	12185.06	15957.91
Instrumental + Confidence	11301.75	25067.15	17405.32	17776	11801.1	15654.25
Instrumental	11312.66	25082.81	17406.1	17768.03	11794.75	15640.29

Supplementary Table 9. *Experiments 1 – 4: R^2 for hypothesized model.*

Variance Test	Experiment 1	Experiment 2, Time 1	Experiment 2, Time 2	Experiment 3, Time 1	Experiment 3, Time 2	Experiment 4
**Conditional R^2**	0.568	0. 429	0. 345	0. 422	0. 515	0. 523
**Marginal R^2**	0. 035	0. 021	0. 035	0. 134	0. 171	0. 089
Participants' Mean R^2	0.234	0.174	0.175	0.294	0.351	0.312

Conditional and Marginal R^2 are calculated for mixed models (Nakagawa & Schielzeth, 2013). Conditional R^2 reflects variability explained by the full model (random and fixed effects). Marginal R^2 reflects the variability explained only by fixed effects, not considering random effects. This is less informative in our case, because of the large individual differences in the weight subjects assign to the different motives, which are captured by random slopes. Participants' mean R^2 represents the average R^2 calculated for each subjects' linear model separately.

Stimuli

All stimuli for Experiments 1, 2, and 5 (traits) were adapted from Allport's trait-word list[1]. Negative stimuli for Experiment 4 (health conditions) were adapted from a WHO report indicating common causes of death[2]. Positive stimuli for Experiment 4 (health conditions) and all stimuli for Experiment 3 (finance questions) were developed by the authors.

Experiment 3, Time 1 stimuli

The following sentences followed the question *"Do you want to know…"*

what your health expenses are?
whether your credit card information has been compromised?
what the Gross Domestic Profit is?
in which percentile income bracket you fall into in your country?
how your salary compares to others doing a similar job?
how much more/less individuals of your gender make in your job relative to the opposite gender?
how much more/less individuals of your race make in your job relative to a different race?
what the gender ratio is for individuals with your job title?
how the Dollar will compare to the Euro at the end of 2020?
how the Dollar will compare to the Yen at the end of 2020?
how much your property is worth?
how much you will pay in travel expenses this year?
how much your phone bill will be this year?
what the unemployment rate is in your country?
what the unemployment rate is in Australia?
what you had spent on dining this year?
the value of gold?
what the financial impact of Brexit is on the global economy?
what your bank balance will be on December 31st, 2020?
what your yearly income will be 5 years from now?
how much each year you will receive from your pension when you retire?
how the stock market will be performing 1 year from now?
whether you will change professions in the future?
what age you will retire?
where you will live 5 years from now?
what your credit score will be in 5 years?
how much your grandparents made when they were younger?
how much your next vacation will cost?
which phone carrier provides the best deal?
whether a family member will get promoted?
whether a family member needs a loan?
how much in total you will spend on utilities in 2021?
how much you will be required to pay in federal and state taxes next year?
what the price of oil and gas will be in 5 years?
the earnings of your favourite celebrity for 2020?
the average value of homes in your neighbourhood?
if you can make more/less income on different platforms from the one you are on now?
what the top financial investments advisors are recommending?
the value of the Dow Jones?
what you had spent on clothing this year?

Experiment 3, Time 2 stimuli

The following sentences followed the question *“Do you want to know…”:*

what Warren Buffet recommends investing in?
the earnings of Donald Trump for 2020?
the value of the NASDAQ?
how much Apple is worth?
how much your hairdresser makes?
how much your water and gas bill will be this year?
what the unemployment rate is in Europe?
what the unemployment rate is in Asia?
which bank gets the best user ratings?
the average cost of a 2-carat diamond?
what the financial impact of Covid-19 is on the global economy?
which airline provides the best deal?
the average value of homes in your city?
if you can make more/less in a different job right now?
how your salary compares to others doing a similar job in a different country?
how much more/less individuals of your race make on average compared to those of different race?
what the gender ratio is for individuals in the bottom 1% of earners?
what the gender ratio is for individuals in the top 1% of earners?
how the US Dollar will compare to the British Pound at the end of 2020?
how the US Dollar will compare to the Canadian Dollar at the end of 2020?
how much your property will be worth in 5 years?
the mortgage rates right now?
how much your assets will be worth in 5 years?
what your yearly income will be next year?
when the next income relief stimulus package will be delivered?
what the inflation rate will be 1 year from now?
whether you will move States for work in the future?
how much you will receive upon retirement?
how large your house will be 5 years from now?
if you will have debt in 5 years?
what your daily expenses are?
how much your next vehicle will cost?
whether a close friend will get promoted?
whether a close friend needs a loan?
how much in total you will spend on commuting in 2021?
whether your bank details have been compromised?
what the national debt is?
how much you will pay for Social Security?
what the price of silver will be in 5 years?
in which percentile income bracket you will fall in 5 years?

Experiment 4

The following sentences followed the question *"Would you want to know if you have a gene that increases your likelihood of…":*

Alzheimer's Disease?
A Youthful Appearance?
Dementia?
Diabetes?
Good Concentration?
Arthritis?
Stroke?
Infertility?
Clear Skin?
Lactose Intolerance?
Strong Immune System?
Liver Disease?
Obesity?
OCD?
Parkinson's Disease?
Long Life Expectancy?
Prostate/Breast Cancer?
Schizophrenia?
Good Hand and Eye Coordination?
Skin Cancer?
Serious Covid-19 Symptoms?
Healthy Cholesterol Level?
Healthy Sleep Cycles?
Brain Tumor?
Fresh Breath?
Good Memory?
Depression?
High Fertility?
High Lung Capacity?
Good Vision?
Heart Disease?
High Intelligence/IQ?
Fast Metabolism?
Leukemia?
Strong Joints?
Being Athletic?
Lung Cancer?
High Tolerance to Stress?
Strong Bones?
Sexual Dysfunction?

Information Avoidance (Experiment 1-4)

Participants indicated they would rather avoid knowledge (that is selected -3, -2, or -1 on the information-seeking question) on 37.83% of the trials in Experiment 1, 23.4% in Experiment 2 Time 1, 30.2% in Experiment 2 Time 2, 23.9% in Experiment 3 Time 1, 28.8% in Experiment 3 Time 2, and 23.5% in Experiment 4.

A paired-samples t-test was conducted to compare the mean ratings on the different scales when individual indicated they preferred knowledge (+3, +2, +1) compared to when they indicated they preferred ignorance (-3, -2, -1). The results are presented in **Supplementary Table 10** below.

Supplementary Table 10. Paired-samples t-test comparing the mean ratings on the different scales between trials on which participants selected knowledge and ones in which they selected to avoid knowledge.

Experiment 1	**Mean Difference Knowledge Trials minus Avoidance Trials (SD)**	**t**	**df**	**p**
Usefulness	0.57 (1.04)	4.450	65	0.0001
Thought Frequency	0.60 (0.97)	5.005	65	0.0001
Feelings to Know	0.70 (0.95)	5.919	65	0.0001
Feelings Never to Know	-0.35 (0.74)	-3.869	65	0.0001
Expected rating of others	0.91 (1.26)	5.862	65	0.0001
Confidence in estimation	0.08 (0.88)	0.778	65	0.439
Experiment 2, Time 1	**Mean Difference Knowledge Trials minus Avoidance Trials (SD)**	**t**	**df**	**p**
Usefulness	0.59 (1.02)	-6.814	137	0.0001
Thought Frequency	0.37 (1.00)	-4.335	137	0.0001
Feelings to Know	0.63 (0.78)	-9.472	137	0.0001
Feelings Never to Know	-0.29 (0.62)	5.583	137	0.0001
Expected rating of others	0.75 (1.04)	-8.502	137	0.0001
Confidence in estimation	0.19 (0.70)	-3.198	137	0.002
Experiment 2, Time 2	**Mean Difference Knowledge Trials minus Avoidance Trials (SD)**	**t**	**df**	**p**

Usefulness	0.56 (0.76)	7.796	110	0.0001
Thought Frequency	0.46 (0.80)	6.015	110	0.0001
Feelings to Know	0.56 (0.75)	7.871	110	0.0001
Feelings Never to	-0.26 (0.56)	-4.938	110	0.0001
Expected rating of others	0.96 (1.12)	9.105	110	0.0001
Confidence in estimation	0.06 (0.65)	0.953	110	0.343

Experiment 3, Time 1	**Mean Difference Knowledge Trials minus Avoidance Trials (SD)**	**t**	**df**	**p**
Usefulness	1.60 (1.33)	12.470	107	0.0001
Thought Frequency	0.75 (0.82)	9.465	106	0.0001
Feelings to Know	1.13 (1.13)	10.368	107	0.0001
Feelings Never to Know	-0.54 (0.59)	-9.606	106	0.0001
Confidence in estimation	0.46 (0.91)	5.264	107	0.0001

Experiment 3, Time 2	**Mean Difference Knowledge Trials minus Avoidance Trials (SD)**	**t**	**df**	**p**
Usefulness	1.60 (1.29)	10.758	74	0.0001
Thought Frequency	1.39 (1.17)	10.298	74	0.0001
Feelings to Know	0.80 (0.70	9.921	74	0.0001
Feelings Never to Know	-0.65 (0.77)	-7.253	74	0.0001
Confidence in estimation	0.37 (0.83)	3.884	74	0.0001

Experiment 4	**Mean Difference Knowledge Trials minus Avoidance Trials (SD)**	**t**	**df**	**p**
Usefulness	1.59<br>(1.34)	11.219	89	0.0001
Thought Frequency	0.73<br>(1.06)	6.520	89	0.0001
Feelings to Know	0.70<br>(0.88)	7.604	89	0.0001
Feelings Never to Know	-0.48<br>(0.77)	-5.954	89	0.0001
Expectations	-0.22<br>(0.95)	-2.192	89	0.031
Confidence in estimation	0.07<br>(0.90)	0.750	89	0.455

Information-seeking Choice and Expectation

Below we plot the distribution of participants' ratings on the question of whether they wanted information in Experiments 1, 2, 3 ,4 and 5. We also plot the distribution of participants' raw ratings on what they thought the information will reveal in Experiments 1, 2, 4 and 5. In Experiment 3 expectations could not be quantified and high/low numbers do not indicate positive/negative expectations. In Experiment 1 and 2, participants' who had more negative expectations of what information was to reveal scored higher on the psychopathology factors (Experiment 1: Anxious-Depression: $r(71) = -0.672$, $p = 0.0001$, Compulsive Behaviour and Intrusive Thought: $r(71) = -0.333$ $p = 0.004$, Social-Withdrawal: $r(124) = -0.571$, $p = 0.0001$; Experiment 2: Anxious-Depression: $r(124) = -0.519$, $p = 0.0001$, Compulsive Behaviour and Intrusive Thought: $r(124) = -0.360$, $p = 0.0001$, Social-Withdrawal: $r(124) = -0.447$, $p = 0.0001$). Information-seeking choice was not significantly correlated with any of the three psychopathology factors in Experiment 1 (Anxious-Depression: $r(80) = -0.094$, $p = 0.434$; Compulsive Behaviour and Intrusive Thought: $r(71) = 0.184$, $p = 0.124$; Social-Withdrawal: $r(71) = -0.051$, $p = 0.672$). In Experiment 2, people who were less likely to want information scored higher on Social-Withdrawal ($r(124) = -0.201$, $p = 0.025$), but not with either Anxious-Depression ($r(124) = -0.007$, $p = 0.939$) nor Compulsive Behaviour and Intrusive Thought ($r(124) = -0.053$, $p = 0.562$).

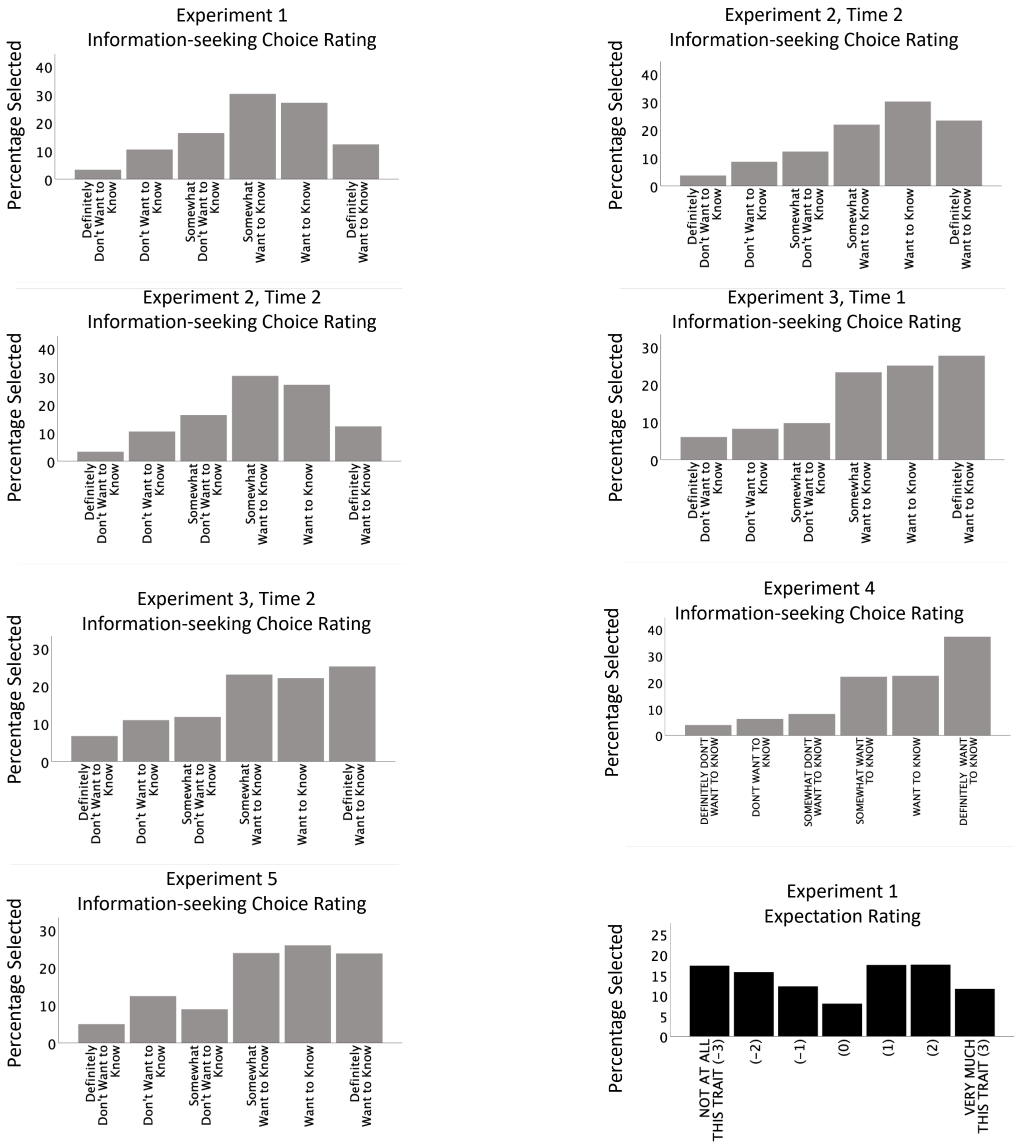

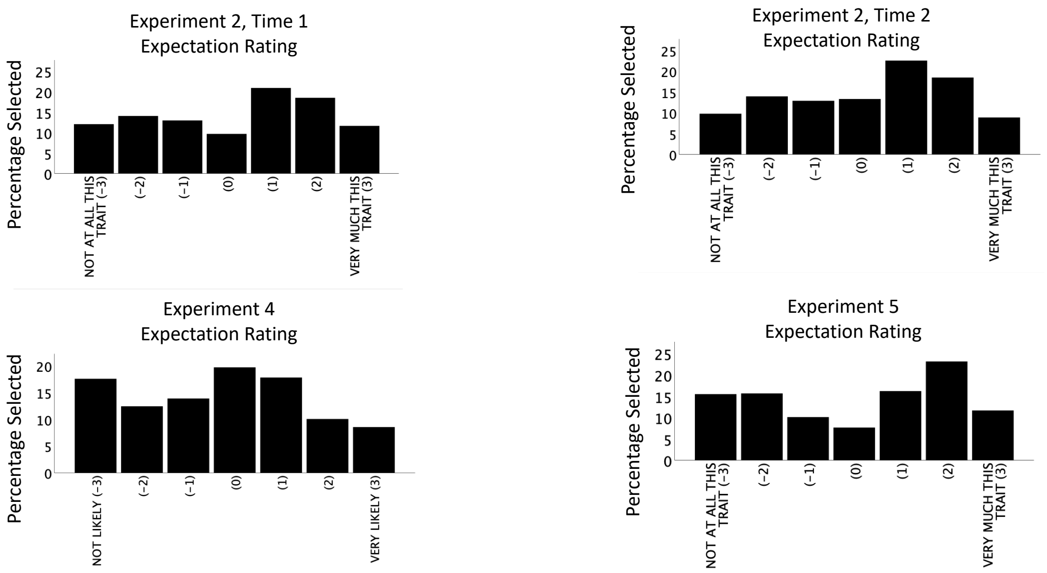

Supplementary Figure 3. Plotted are the distributions of participants' ratings on the question of whether they wanted information (grey) in Experiments 1, 2, 3, 4, and 5 and participants' ratings on what they thought the information will reveal (black) in Experiments 1, 2, 4 and 5.

Supplementary Table 11. Experiments 1, 2 & 4: Substituting Hedonic Utility for the mean of Hedonic Utility and Rating of Expected Information in the theorized model (provided as response to a reviewer's request).

	**B (SE)**	**df**	**t-value**	**p (two-sided)**
**Experiment 1**				
Instrumental Utility	0.106 (0.03)	55.35	3.800	0.0004
Mean of Hedonic Utility & Expectations	0.175 (0.03)	70.99	6.086	0.0001
Cognitive Utility	0.095 (0.03)	91.79	3.011	0.0003
**Experiment 2, Time 1**				
Instrumental Utility	0.076 (0.02)	160.29	4.360	0.0001
Mean of Hedonic Utility & Expectations	0.180 (0.02)	165.02	8.112	0.0001
Cognitive Utility	0.057 (0.02)	171.52	3.729	0.0003
**Experiment 2, Time 2**				
Instrumental Utility	0.084 (0.02)	79.44	4.311	0.0001
Mean of Hedonic Utility & Expectations	0.216 (0.03)	108.95	8.318	0.0001
Cognitive Utility	0.092 (0.02)	127.05	4.963	0.0001
**Experiment 4**				
Instrumental Utility	0.248 (0.03)	123.27	9.346	0.0001
Mean of Hedonic Utility & Expectations	0.080 (0.02)	121.52	3.435	0.0008
Cognitive Utility	0.115 (0.02)	135.16	7.639	0.0001

Note. Raw scores for Expectations were reverse scored for negative stimuli. Raw scores were then centered within participants and entered into a linear mixed effect model with a random intercept and slope for subject and random intercept for item.

Supplementary References

1. Allport, G. W., & Odbert, H. S. (1936). Trait-names: A psycho-lexical study. *Psychological monographs*, *47*(1), i.

2. World Health Organization. (2018). WHO methods and data sources for country-level causes of death 2000-2016. *Global Health Estimates Technical Paper WHO/HIS/IER/GHE/2018.3; World Health Organization: Geneva, Switzerland*.
