# Supplementary material for: Individual differences in information-seeking
Source: Nat Commun. 2021 Dec 3;12:7062. doi: 10.1038/s41467-021-27046-5 (PMC8642448; doi:10.1038/s41467-021-27046-5)
Supplement: Supplementary file 3 — Reporting summary [file 41467_2021_27046_MOESM3_ESM.pdf]

nature research

Corresponding author(s): Tali Sharot, Christopher Kelly

Last updated by author(s): October 22nd, 2021

Reporting Summary

Nature Research wishes to improve the reproducibility of the work that we publish. This form provides structure for consistency and transparency in reporting. For further information on Nature Research policies, see Authors & Referees and the Editorial Policy Checklist .

Statistics

For all statistical analyses, confirm that the following items are present in the figure legend, table legend, main text, or Methods section.

n/a	Confirmed	
☐	☒	The exact sample size (*n*) for each experimental group/condition, given as a discrete number and unit of measurement
☐	☒	A statement on whether measurements were taken from distinct samples or whether the same sample was measured repeatedly
☐	☒	The statistical test(s) used AND whether they are one- or two-sided<br>*Only common tests should be described solely by name; describe more complex techniques in the Methods section.*
☐	☒	A description of all covariates tested
☐	☒	A description of any assumptions or corrections, such as tests of normality and adjustment for multiple comparisons
☐	☒	A full description of the statistical parameters including central tendency (e.g. means) or other basic estimates (e.g. regression coefficient) AND variation (e.g. standard deviation) or associated estimates of uncertainty (e.g. confidence intervals)
☐	☒	For null hypothesis testing, the test statistic (e.g. *F*, *t*, *r*) with confidence intervals, effect sizes, degrees of freedom and *P* value noted<br>*Give P values as exact values whenever suitable.*
☒	☐	For Bayesian analysis, information on the choice of priors and Markov chain Monte Carlo settings
☒	☐	For hierarchical and complex designs, identification of the appropriate level for tests and full reporting of outcomes
☐	☒	Estimates of effect sizes (e.g. Cohen's *d*, Pearson's *r*), indicating how they were calculated

Our web collection on statistics for biologists contains articles on many of the points above.

Software and code

Policy information about availability of computer code

Data collection	The experiments were designed using Qualtrics' online survey platform. Participants were recruited using Prolific and Amazon Mechanical Turk.
Data analysis	IBM SPSS 27, R studio (Version 1.3.1056)

For manuscripts utilizing custom algorithms or software that are central to the research but not yet described in published literature, software must be made available to editors/reviewers. We strongly encourage code deposition in a community repository (e.g. GitHub). See the Nature Research guidelines for submitting code & software for further information.

Data

Policy information about availability of data

All manuscripts must include a data availability statement. This statement should provide the following information, where applicable:

- Accession codes, unique identifiers, or web links for publicly available datasets
- A list of figures that have associated raw data
- A description of any restrictions on data availability

Anonymized behavioral data are available on GitHub (github.com/affective-brain-lab/Deciding_what_to_know_2020).

Field-specific reporting

Please select the one below that is the best fit for your research. If you are not sure, read the appropriate sections before making your selection.

☐ Life sciences ☒ Behavioural & social sciences ☐ Ecological, evolutionary & environmental sciences

Behavioural & social sciences study design

All studies must disclose on these points even when the disclosure is negative.

Study description	This study investigated different motives of information-seeking and how they relate to mental health. Experiments 1, 4 & 5 were quantitative cross-sectional studies. Experiments 2 & 3 were longitudinal studies.
Research sample	Our research sample included participants recruited from Prolific and Amazon Mechanical Turk: Experiment 1 (age = 37.69, SD = 9.18; females = 46.3%), Experiment 2, Time 1 (age = 28.00, SD = 9.66; females = 47.2%), Experiment 2, Time 2 (age = 26.93 SD = 8.30; females = 46.00%), Experiment 3, Time 1 (age = 31.91, SD = 9.76; females = 46.7%), Experiment 3, Time 2 (age = 32.88, SD = 9.86; females =52.4%), Experiment 4, (age = 31.15, SD = 11.30; females =56.9%), Experiment 4, (age = 31.85, SD = 10.47; females =43.8%). This allowed us to recruit a large random sample of participants. Our sample is not necessarily representative of the general population.
Sampling strategy	For Experiment 1, sample size was calculated based on a pilot study.<br>For Experiment 2, sample size was based on the effect size of mental health effect in experiment 1.<br>For Experiment 3, sample size was based on the effect size of the longitudinal result in experiment 2.<br>For Experiments 4&5, sample size was based on the effect size of Experiment 1's linear mixed effect model.<br>Power analysis for Experiments 1-5 was conducted using G*power (http://www.psychologie.hhu.de/arbeitsgruppen/allgemeinepsychologie-<br>und-arbeitspsychologie/gpower.html), with .95 power and !=0.05.<br>All experiments implemented a convenient sampling strategy (online participants).
Data collection	All data was collected online using Qualtrics' survey platform by Christopher Kelly. All data was anonymised. No blinding was necessary as there was only one condition.
Timing	Data collection for Experiment 1 took place on 2/11/2019; Experiment 2, Time 1 took place on 18/1/2020; Experiment 2; Time 2 took place between 9/02/2020 and the 13/03/2020; Experiment 3, Time 1 took place on 28/07/2020; Experiment 3, Time 2 took place between 13/08/2020 and the 18/08/2020; Experiment 4 took place on 3/02/2021; Experiment 5 took place on 30/06/2020.
Data exclusions	Experiment 1: 3 participants failed attention checks; 16 participants gave the same exact response on all trials in at least one of the task ratings and thus their beta coefficients could not be calculated.<br>Experiment 2, Time 1: 1 participant failed the attention check; 10 gave the same exact response on all trials for at least one of the task ratings and thus their beta coefficients could not be calculated.<br>Experiment 2, Time 2: 4 participants failed the attention check; 9 gave the same exact response on all trials for at least one of the task ratings and thus their beta coefficients could not be calculated.<br>Experiment 3, Time 1: 8 participants failed the attention check; 19 gave the same exact response on all trials for at least one of the task ratings and thus their beta coefficients could not be calculated.<br>Experiment 3, Time 2: 1 participants failed the attention check; 10 gave the same exact response on all trials for at least one of the task ratings and thus their beta coefficients could not be calculated. 2 participants were not included due to providing different Prolific ID's for each time point.<br>Experiment 4: 4 participants failed the attention check; 28 gave the same exact response on all trials for at least one of the task ratings and thus their beta coefficients could not be calculated.<br>Experiment 5: 1 participant failed the attention check; 2 gave the same exact response on all trials for at least one of the task ratings and thus their beta coefficients could not be calculated.
Non-participation	No participants declined to participate or dropped out.
Randomization	The experiments focused on within-subjects effects and no randomization into groups was required.

Reporting for specific materials, systems and methods

We require information from authors about some types of materials, experimental systems and methods used in many studies. Here, indicate whether each material, system or method listed is relevant to your study. If you are not sure if a list item applies to your research, read the appropriate section before selecting a response.

Materials & experimental systems

n/a	Involved in the study
☒	☐ Antibodies
☒	☐ Eukaryotic cell lines
☒	☐ Palaeontology
☒	☐ Animals and other organisms
☐	☒ Human research participants
☒	☐ Clinical data

Methods

n/a	Involved in the study
☒	☐ ChIP-seq
☒	☐ Flow cytometry
☒	☐ MRI-based neuroimaging

Human research participants

Policy information about studies involving human research participants

Population characteristics	See above.
Recruitment	Participants were recruited using Amazon Mechanical Turk and Prolific's online recruitment platforms. The purpose of this study was not mentioned in the advertisement of the study.
Ethics oversight	Experiments were approved by the ethics committee at UCL and all subjects gave informed consent.

Note that full information on the approval of the study protocol must also be provided in the manuscript.
